# Type 2 diabetes mellitus patients’ knowledge, attitude and practice of lifestyle modifications

**DOI:** 10.4102/hsag.v27i0.1921

**Published:** 2022-10-21

**Authors:** Paul I. Peter, Wilhelm J. Steinberg, Cornel van Rooyen, Johan Botes

**Affiliations:** 1 Department of Family Medicine, Faculty of Health Sciences, University of the Free State, Bloemfontein, South Africa; 2 Department of Biostatistics, Faculty of Health Sciences, University of the Free State, Bloemfontein, South Africa

**Keywords:** diabetes mellitus, type 2, lifestyle modifications, knowledge, attitude, practice, barriers

## Abstract

**Background:**

Type 2 diabetes mellitus (T2DM) is a significant health burden globally, with uncontrolled DM often resulting in short- and long-term complications. Unfortunately, healthcare providers have little control over patients’ necessary lifestyle modification practices outside the consultation room.

**Aim:**

To determine the level of knowledge, attitude and practice (KAP) of lifestyle modifications among patients with T2DM attending the National District Hospital Outpatient Department, Bloemfontein, and identify possible barriers to lifestyle modifications experienced by patients.

**Setting:**

Outpatient Department at a Free State hospital.

**Methods:**

Using a cross-sectional study, patients with previously diagnosed T2DM were invited to participate. Respondents completed a structured questionnaire to assess their KAP regarding lifestyle modifications.

**Results:**

Of the 149 respondents, 64.4% (*n* = 94) were obese and 24.0% (*n* = 35) overweight despite good knowledge of lifestyle modifications. Respondents displayed a positive attitude toward physical exercise but less so to adjusting their diets. In practice, 63.4% (*n* = 83) claimed to exercise regularly, but two-thirds irregularly monitored their weight. The practice of a controlled and planned diet was poor. Perceived barriers to lifestyle modifications included being too busy to engage in physical exercise, bad weather and financial constraints.

**Conclusion:**

Despite displaying good knowledge regarding lifestyle modifications, the attitude and practice thereof remain poor. It is essential to re-emphasise to patients why it is crucial to engage in lifestyle modification practices and assess whether they are doing so correctly.

**Contribution:**

It highlights the importance of lifestyle considerations of T2DM patients in the clinical context.

## Introduction

Diabetes mellitus (DM) is defined as a metabolic disorder where an insulin deficiency results in an excess of glucose (chronic hyperglycaemia), which has a damaging effect on normal metabolic processes (World Health Organization [WHO] 2019). According to the International Diabetes Federation (IDF), an estimated 9.3% of adults between the ages of 20 and 79 years worldwide are currently affected by metabolic disease. Projections estimate that by 2045, the number will have increased from the current 463 million to 700 million (IDF 2019:34).

Type 2 diabetes mellitus (T2DM) occurs as a result of the individual having insulin resistance or relative insulin deficiency. It involves an interplay of interaction between genetic, environmental and behavioural risk factors. The disease has an insidious onset and may remain undiagnosed for many years (Kiberenge et al. 2010; Yoon et al. 2006). The short-term complications of uncontrolled DM include hypoglycaemia and hyperosmolar hyperglycaemic nonketotic syndrome. The long-term complications develop over the years as a result of the damages to the blood vessels (American Diabetes Association [ADA] 2019).

The impact of T2DM in Africa, and most other developing countries, has reached alarming proportions. A staggering 79.4% of adults with DM live in low- and middle-income countries. Even more troubling is that 59.7% of people with DM from Africa are still unaware they have this chronic disease. The African region is also estimated to have the highest percentage (73.1%) of DM-related deaths under 60 years (IDF 2019:34, 43, 54).

Several lifestyle factors play a prominent role in developing T2DM, such as diet, sedentary lifestyle, smoking and alcohol consumption (ADA 2019; Cullmann, Hilding & Östenson 2012; Wu et al. 2014). Lifestyle modification is the foundation of T2DM control, but often little is known about its application and practice among patients (Hu 2011).

More than 80% of all adults with DM are overweight, and nearly half are obese (Nguyen et al. 2011). Diet plays a crucial role in developing T2DM, and it is considered a modifiable risk factor (Wu et al. 2014). Because of the relationship between body weight and insulin resistance, weight loss has been a long-standing recommended strategy for overweight DM patients (ADA 2014). Still, many DM patients often experience finding the appropriate food challenging and consider adherence to an ideal food plan unachievable (ADA 2017). Behavioural change is one of the main obstacles health professionals faces with DM patients (Evert et al. 2014).

Sedentary behaviours, such as inactivity and sitting at work or social gatherings, increase the risk of T2DM. Increased mechanisation and driving have also displaced physical activity in industrialised nations. This trend is on the increase in developing countries (Hu 2011). Television watching has been identified as a significant risk factor. Exercise improves blood glucose control in T2DM, diminishes cardiovascular risk factors, assists with weight loss and promotes well-being (Chen et al. 2015; Colberg et al. 2016; Lin et al. 2015; Puepet et al. 2007).

Another modifiable risk factor for T2DM is cigarette smoking. Current smokers have a 45% higher risk of developing DM compared with nonsmokers. Smoking is associated with an increased risk of truncal obesity or abdominal fat, which is an established risk factor for insulin resistance and DM (Maddatu et al. 2017; Willi et al. 2007).

There is a reduced risk of developing DM among light and moderate alcohol consumers, with a reportedly 30% – 40% lower risk among those consuming one to two drinks per day compared with heavy drinkers or nondrinkers. However, the risk of DM among those who consumed three or more drinks per day was similar to the risk of abstainers (Hu 2011; Koppes et al. 2005).

Lifestyle modifications help improve the effectiveness of pharmacologic intervention. In some cases, the changes lead to a reduction in the required dosages of antidiabetic medications. The proposed lifestyle interventions include educational support and improvement in diet and level of physical activity (Chen et al. 2015; Evert et al. 2014).

Knowledge is a powerful tool in the fight against DM. Educative information can help people better understand DM risks and motivate them to seek proper treatment and take care of their condition (Puepet et al. 2007). In a study carried out in Iran, only 2% of the respondents had good knowledge of DM. Results showed a low awareness of lifestyle modifications regarding weight management (Mohammadi et al. 2015). In contrast, an Ethiopian study by Adem et al. (2014) found that 77% of participants displayed adequate knowledge. A similar study in South Africa reported 92.1% of the respondents had poor knowledge regarding the benefits of exercise and weight loss, and 73.3% had poor knowledge regarding a healthy diet (Okonta, Ikombele & Ogunbanjo 2014). These studies found that attitudes towards lifestyle modifications tended to be positive, especially when knowledge was sufficient (Adem et al. 2014; Mohammadi et al. 2015; Okonta et al. 2014).

Even with patients being aware of or adopting lifestyle modifications, adhering to the recommendations remains a challenge. Patients with chronic conditions have a lower adherence rate to treatment than acute cases, and the decline in adherence is often significant after the first 6 months of commencing therapy (Puepet et al. 2007). This may also be the case with lifestyle adjustments. Mumu et al. (2014) listed several contributing factors to poor adherence: socio-economic issues, the patient’s age, how long the patient has lived with the disease, lack of communication with the healthcare provider, negative health beliefs and unbridgeable views regarding the necessary modifications (Mumu et al. 2014).

### Aim

This study aimed to determine the level of knowledge, attitude and practice (KAP) of lifestyle modifications with regard to diet and exercise among the T2DM patients attending the Outpatient Department (OPD) at the National District Hospital, Bloemfontein. Furthermore, the authors looked to identify the barriers to regular physical exercises and adherence to recommended dietary plans.

## Methods

### Study design and setting

This was a cross-sectional study. The researchers chose the OPD of the National District Hospital as the setting for this project.

National District Hospital is a level one public hospital that provides various medical services to the residents of Bloemfontein and neighbouring towns. The OPD mainly manages and follows up with patients with chronic medical conditions, including T2DM and hypertension. An estimated 1500 patients are seen every month.

### Study population and sampling strategy

The study population included patients attending the OPD who had been diagnosed with T2DM for more than a year before the study. Patients who were unable to answer the questionnaire and those who did not consent to participate were excluded.

A systematic sampling method was used in the selection of the participants. The estimated sample size over the 3-month study period, based on the number of patients attending the OPD, was 218 patients. Every second consenting patient who satisfied the inclusion criteria was included. The study period was from March 2019 to June 2019.

### Data collection

A structured questionnaire was developed based on the objectives of the study and topic-relevant literature. The questionnaire was developed in English but also translated by a staff member into Sesotho and Afrikaans – the most common languages spoken in the region. The questionnaire would be self-administered, but a research assistant was available when needed.

The questionnaire was divided into seven sections, mainly consisting of closed-ended questions. The first three sections captured data on the respondents’ socio-demographic characteristics (ages, gender, marital status, educational level, employment status, smoking status and alcohol use), number of years since diagnosis of T2DM, general knowledge about the disease and current mode of treatment. It also included the measurements of physical attributes such as the weight and height of the respondents to calculate their body mass index (BMI).

Section D comprised eight questions which included the respondents’ grading of their knowledge of lifestyle modification practices, sources of information on lifestyle modifications and assessment of respondents’ knowledge of lifestyle modification practices. Section E captured information on the attitudes of the respondents to lifestyle modification practices. A 5-point Likert scale was used to assess the attitudes of the respondents to regular physical exercise, adjustment of their diet and weight control. Section F assessed the extent of the practice of lifestyle modifications. The last section aimed at identifying the barriers to regular physical exercises and adherence to recommended dietary plans among the respondents.

### Pilot study

A pilot study was conducted in September 2018 by administering 10 questionnaires to diabetic patients at the OPD. Unclear questions were identified and amended. The content validity was confirmed, as the questions were relevant to the objectives and clear. Data from the pilot study were excluded from the study.

### Data analysis

Data were analysed by the Department of Biostatistics, Faculty of Health Sciences, University of the Free State, using the statistical analysis software SAS 9.4 (SAS Institute, Cary, North Carolina, United States). Descriptive statistics, including the frequency, percentages, means and standard deviation were used to determine the distribution of the variables. Categorical variables such as gender, marital status and educational level were summarised by frequency and percentages. Continuous variables such as age, weight, height and BMI were summarised by medians, minimum, maximum or percentiles and described with frequency and percentages. Significance was set at *p* < 0.05.

### Ethical considerations

Ethical approval for this study was obtained from the Health Sciences Research Ethics Committee, Faculty of Health Sciences of the University of Free State (reference number UFS-HSD2017/0772). Permission to conduct the study at National District Hospital was granted by the Free State Department of Health. Respondents gave written informed consent before they were enrolled in the study.

To protect confidentiality, unique numbers were given to each questionnaire, and no identifiable information was recorded. Questionnaires were collected by the research assistants and professional nursing personnel, and stored safely until the first author collected and captured the data. The data were captured and stored on a password-protected computer.

## Results

Out of the estimated sample size of 218, 150 respondents consented to participate in the study (response rate 68.8%). One questionnaire was labelled unsuitable for inclusion, and 149 respondents were included in the analysis.

The mean age of the 137 (91.9%) respondents who indicated their age was 56 years (range 22–81 years). Almost 70% of the respondents were women (69.8%), and 50.0% were married, while 23.0% were single. The highest percentage of respondents completed some level of secondary education (43.2%), followed by 25.9% who matriculated, while 42.6% were pensioners, 24.3% were employed and 24.3% unemployed. A quarter (22.2%) of the respondents were currently smoking, and 26.3% reported currently drinking. According to the BMI, 64.4% of the respondents were obese (Table 1).

**TABLE 1:** Sociodemographics and type 2 diabetes mellitus history of respondents.

Variable	*n*	%
**Gender (*n* = 149)**		
Male	45	30.2
Female	104	69.8
**Marital status (*n* = 148)**		
Single	34	23.0
Married	74	50.0
Divorced	13	8.8
Widowed	26	17.6
Cohabiting	1	0.7
**Highest level of education (*n* = 139)**		
Completed some primary school	25	18.0
Completed primary school	6	4.3
Completed some secondary school	60	43.2
Matriculated (Grade 12)	36	25.9
Tertiary level education	12	8.6
**Employment status (*n* = 148)**		
Employed	36	24.3
Unemployed	36	24.3
Pensioner	63	42.6
Housewife	12	8.1
Student	1	0.7
**Currently smoking (*n* = 149)**		
Yes	33	22.2
**Currently drinking alcohol (*n* = 148)**		
Yes	39	26.4
**Body mass index (kg/m^2^) (*n* = 146)**		
Normal (18.5 kg/m^2^ – 24.9 kg/m^2^)	17	11.6
Overweight (25.0 kg/m^2^ – 29.9 kg/m^2^)	35	24.0
Obese (≥ 30.0 kg/m^2^)	94	64.4
**Years since T2DM diagnosis (*n* = 148)**		
1–3	38	25.7
4–6	21	14.2
7–9	16	10.8
≥ 10	73	49.3
**Current treatment for DM (*n* = 148)**		
Oral hypoglycaemic	87	58.8
Insulin	37	25.0
Combination of insulin and oral hypoglycaemic	22	14.9
Other	2	1.4

DM, diabetes mellitus; T2DM, type 2 diabetes mellitus.

### Diagnosis and current treatment

As shown in Table 1, for half (49.3%) of the respondents, it had been more than 10 years since their DM diagnosis, while 25.7% were diagnosed between 1 to 3 years prior. Almost 60.0% of the respondents’ current treatment included only an oral hypoglycaemic, while 14.9% were on a combination of insulin and an oral hypoglycaemic.

### Knowledge of type 2 diabetes mellitus

On the question on possible causes of T2DM, 56 (37.6%) respondents stated a lack of insulin could cause DM, while 13 (8.7%) indicated that inadequate insulin production could also lead to the disease. Thirty-nine (26.2%) respondents selected being overweight, and 70 (47.0%) eating unhealthy food as possible causes for DM. Seven (4.7%) thought that drinking too much water could result in DM.

One question attempted to ascertain whether respondents knew what they could do to improve their sugar levels. Most (*n* = 103, 69.1%) stated that eating a healthy diet could improve blood sugar levels. Half (*n* = 75, 50.3%) knew that regular exercise could improve blood sugar levels, while 53 (35.6%) understood that weight control could be beneficial. A total of 51 (34.2%) respondents believed that a patient’s blood sugar level could only be improved by taking medication. When asked whether they had ever heard of the term ‘HbA1c’ or ‘glycated haemoglobin’, 125/145 (86.2%) responded ‘No’.

### Knowledge of lifestyle modifications

The authors asked respondents to grade their own perceived knowledge of lifestyle modification practices recommended for people with DM. Six (4.2%) respondents stated they had excellent knowledge, 40 (27.8%) good, 82 (56.9%) average and 16 (11.1%) said they considered their knowledge poor.

When asked who informed them about lifestyle modification practices, 141 (94.6%) respondents indicated healthcare personnel, 11 (7.4%) from the media, while five (3.4%) said their relatives and friends were their sources.

The respondents were asked to indicate what they understand of lifestyle modification practices. Sixteen (10.7%) respondents indicated healthy dietary habits only, and nine (6.0%) stated regular physical exercise. In comparison, 130 (87.3%) respondents indicated that lifestyle modification practices included both healthy dietary habits and regular physical exercise.

When the respondents were asked to indicate how much time a patient with DM should exercise daily, 10 (6.8%) said < 15 min per day, 17 (11.6%) < 30 min per day, 90 (61.2%) said at least 30 min per day, while 30 (20.4%) did not know how long a DM patient should exercise.

As summarised in Table 2, most respondents (70.1%) agreed that patients with DM should eat at least three evenly spaced meals per day. Almost all felt that patients with DM should limit their fat intake (94.6%), that they should limit intake of refined sugar (89.1%) and that low-fat milk is better than full cream (88.3%).

**TABLE 2:** Knowledge of a healthy diet for patients with type 2 diabetes mellitus.

Statement	Agree		Disagree		Not sure	
	*n*	%	*n*	%	*n*	%
Should eat at least three evenly spaced meals (*n* = 147)	103	70.1	23	15.7	21	14.3
Should limit fat intake (*n* = 147)	139	94.6	6	4.1	2	1.4
Should limit intake of refined sugar (*n* = 147)	131	89.1	4	2.7	12	8.2
Low-fat milk is better than full cream (*n* = 145)	128	88.3	10	6.9	7	4.8

### Attitude towards lifestyle modifications

As summarised in Table 3, almost all the respondents agreed to strongly agreed that engaging in physical exercise (94.6%) is important and that controlling one’s weight (94.5%) is vital. However, 56.5% disagreed to strongly disagreed that modifying one’s diet is essential.

**TABLE 3:** Attitude towards the importance of lifestyle modifications.

Variable	Strongly disagree		Disagree		Not sure		Agree		Strongly agree	
	*n*	%	*n*	%	*n*	%	*n*	%	*n*	%
Physical exercise (*n* = 147)	2	1.4	1	0.7	5	3.4	129	87.8	10	6.8
Diet modification (*n* = 147)	5	3.4	78	53.1	11	7.5	45	30.6	8	5.4
Weight control (*n* = 146)	5	3.4	0	0.0	3	2.1	127	87.0	11	7.5

### Practice of lifestyle modifications

In Table 4, the actual practice of lifestyle modifications is indicated. Most (133/148, 89.9%) stated they engage in some form of physical exercise. Of these 133 respondents, 72.2% engaged in brisk walking or other physical activity (21.1%), such as weight lifting. The majority (107/131, 81.7%) of the respondents’ duration per session was ≥ 30 min, followed by 20–30 min for 16 (12.2%) respondents. Frequency of exercise was daily (83/131, 64.4%), followed by at least three times a week (*n* = 35, 26.7%).

**TABLE 4:** Practice of lifestyle modifications.

Practice	*n*	%
**Engage in physical exercise (*n* = 133)**		
Brisk walking	96	72.2
Cycling	8	6.0
Jogging	16	12.0
Sport activities	7	5.3
Gardening or farming	15	11.3
Other (e.g. physiotherapy, weight lifting)	28	21.1
**Follow a controlled and planned diet (*n* = 80)**		
High starch and fibre diets	4	5.0
Low saturated fat and calorie intake	27	33.8
Fruits and vegetables	73	91.3
Eat any kinds of food	4	5.0
**Monitor weight regularly (*n* = 146)**	**52**	**35.6**

Just more than half (80/142, 56.3%) said they follow a controlled and planned diet. Most (91.3%) of the respondents listed the intake of fruits and vegetables as part of their diet. None of the respondents selected regulating alcohol intake as part of a controlled and planned diet. Two-thirds (53, 66.3%) of the respondents followed these dietary recommendations at least three times a week. Only a third (35.6%) checked their weight regularly.

### Possible barriers to lifestyle modifications

The authors set out to identify barriers that may prevent T2DM patients from engaging in lifestyle modifications. The respondents were asked to share their opinions on what barriers may cause them to neglect physical exercise or adhere to the recommended dietary plan (Table 5).

**TABLE 5:** Possible barriers to physical exercise and recommended dietary plan (*n* = 149).

Lifestyle modification	*n*	%
**Reasons for not engaging in physical exercise**		
Too busy schedule	61	40.9
Bad weather	45	30.2
Lacking exercise partner	7	4.7
Criticism	4	2.7
Too old	11	7.4
Lack of exercise facilities	4	2.7
Poor health	8	5.4
Other	38	25.5
**Reasons for not adhering to recommended dietary plan**		
I am always eating out	3	2.0
Financial constraints (cannot afford the health diets)	74	49.7
I have poor self-control	20	13.4
Situations at home (family eat from a common pot at home)	22	14.8
Lack of information on proper diet	7	4.7
Lack of support at home	1	0.7
Other	19	12.8

The highest percentage of respondents noted that a busy schedule (40.9%) and bad weather (30.2%) are barriers to physical exercises. A low percentage noted age (7.4%) and poor health (5.4%) as barriers. Other reasons included poor vision and work-related issues.

Half (49.7%) of the respondents cited financial constraints as a reason for not adhering to the recommended dietary plan, while 14.8% noted circumstances at home, such as sharing a meal from one pot. Other reasons included sitting at home. One respondent stated that they did not always have the food they needed to eat.

Because of the smaller than intended number of respondents, no significant associations were made between the sociodemographic characteristics, BMI and KAP regarding lifestyle modifications.

## Discussion

Almost half of the respondents (49.3%) were diagnosed with T2DM more than 10 years before the study. Patients who lived with DM for an extended period, combined with hypertension and poor glycaemic control, tend to experience an onset of microvascular complications (Ramanathan 2017). Thus, many of these respondents will benefit from regular screening for DM-related complications, for example testing visual acuity and screening for peripheral neuropathy.

The majority of the respondents showed poor knowledge regarding the cause of DM, with only 26.2% stating overweight as a causative factor. Most respondents understood the importance of regular physical exercise and a healthy diet in improving their overall health, even though only 35.6% understood that weight control could improve their blood sugar levels. Optimal management of the metabolic disorder encompasses adoption and adherence to the combination of prescribed drug therapy, dietary modifications, regular physical exercises and other self-care activities. This requires having adequate knowledge and understanding of the disease, displaying a good attitude and bringing the lifestyle modifications to practice.

### Knowledge, attitude and practice of lifestyle modifications

More than half (56.9%) of the respondents considered their knowledge of lifestyle modification practices as average. The majority (94.6%) of the respondents were informed about lifestyle modifications from healthcare personnel. Compared to the Ethiopian study by Adem et al. (2014) that recorded 58.6%, this result could highlight a successful awareness drive from healthcare services.

Most (87.3%) agreed that lifestyle modification practices included both healthy dietary habits and regular physical exercises. This is almost double to a similar study conducted in Botswana that recorded 48.1% (Ganiyu et al. 2013). Two-thirds (61.2%) of the respondents were aware that T2DM patients should engage in physical exercise for at least 30 min per day. In comparison, a study from Sri Lanka found that most of the participants did not understand this requirement (Ranasinghe et al. 2015). Regarding healthy dietary habits, respondents seem to be aware that they should eat at least three evenly spaced meals per day but also that they should limit their fat and refined sugar intake.

The good knowledge of lifestyle modification practices among the respondents could be explained by higher than expected literacy level. A similar project in Gauteng (Okonta et al. 2014) reported no more than 6.6% participants achieving higher than primary school, while most (77.7%) of the respondents in this study’s sample completed some secondary school training or higher. Several factors influence the level of knowledge, such as literacy level, training received, availability of information and the period patients have lived with the disease (Babikr et al. 2017; Kiberenge et al. 2010).

Almost all the respondents displayed a positive attitude towards physical exercise. Unfortunately, it seems they did not share the same enthusiasm for dietary adjustments. Almost half of the respondents noted financial constraints and the inability to afford the health diets as a barrier. Despite this, an overwhelming majority (94.5%) in this study showed positivity towards the need for stringent weight control. But again, only a third checked their weight regularly.

Unfortunately, good knowledge and attitude do not seem to carry over to the necessary practice. More than half stated they were attempting to follow a controlled and planned diet, but the general practice of dietary modification was poor. This may not come as a surprise, considering that more than half of the respondents showed a poor attitude towards dietary modification. Half of the respondents reported financial constraints to be the reason for nonadherence to the recommended dietary plan. This could be because of the respondents being either unemployed or pensioners. Financial constraints may also explain the respondents’ earlier reported poor attitude to dietary modification because adjusting their diet might further strain their pockets. Financial difficulties and food insecurity as well as the cost of appropriate foods are all well-known barriers to following dietary recommendations (Muchiri, Gericke & Rheeder 2012).

Although almost all respondents stated that they engage in exercise – most of whom indicated brisk walking – only 63.4% engaged in regular physical exercise. In an attempt to discover which factors act as barriers for T2DM patients, an obstruction seems to be being overweight. This is hardly surprising considering that two-thirds of the respondents presented as obese. Ball et al. (2000) reported that feeling ‘too fat to exercise’ is a common problem among overweight patients, particularly women. Thirty per cent of the respondents reported bad weather as a barrier to engaging in physical exercise.

Despite showing decent knowledge and even attitudes – bar the dietary modification – the actual practice of lifestyle modifications is insufficient. These, together with obese results from the BMI measurement, do not automatically follow into practice. This could mean that making patients aware of lifestyle modifications is not enough to motivate them.

### Limitations

Even though the small sample size does not allow the findings to be generalisable, the authors believe the discussion of compliance to lifestyle modification should be revisited. Selection of participants (as it was only one health facility), language barriers and differing cultural viewpoints may have influenced some outcomes. As the questionnaire was self-developed, the reliability has not been established.

## Conclusion

The study showed that even though most respondents displayed adequate knowledge of lifestyle modification practices, many still have a poor attitude towards making the necessary changes, and few adapted their way of life. Seeing that most of the respondents presented either as overweight or obese, these results are especially troublesome.

The barriers stated by the respondents to implementing lifestyle modifications included being too busy, financial constraints, poor self-discipline and even bad weather.

There is a need to address the poor attitude to dietary modification and the suboptimal engagement in physical exercise among these patients. The importance of correctly engaging in lifestyle modification practices needs to be regularly emphasised by healthcare professionals. Continuous support and motivation are required to improve compliance to reach optimal control of their blood glucose; in doing so, it will help prevent potential complications.

### Recommendations

Patients with T2DM should be encouraged to participate in facility-organised multidisciplinary DM self-management education programmes. These programmes are beneficial for facilitating the necessary knowledge and skills. Diabetic patients should be encouraged to share their challenges regarding lifestyle modification practices with health professionals and even other patients within support group meetings.

The importance of healthy dietary habits and regular physical exercise must be continuously emphasised, especially to overweight or obese patients. Many of the patients will benefit from brief behavioural change counselling; therefore, the doctors and other healthcare professionals attending to these patients must receive training on this essential skill. Since completion of the project, medical practitioners working at the health facility attended brief lectures on behavioural counselling techniques.

Other healthcare team members should be included and frequently consulted in an attempt to improve compliance. Dietitians can assist patients in identifying affordable meal plans, while psychologists, occupational therapists and social workers all play roles in helping with patients’ motivation.
